# Research on the Preparation of Porous Ceramics from Gold Tailings and the Thermal Insulation and Heat Resistance Properties

**DOI:** 10.3390/ma18204764

**Published:** 2025-10-17

**Authors:** Haoyu Zhao, Hongzhi Yue, Jianping Zhu, Laijun Ma, Jiayi Zhong, Wenjuan Jiao, Yan Wang, Zhiyang Chang

**Affiliations:** School of Materials Science and Engineering, Shandong University of Technology, Zibo 255000, China15695400876@163.com (J.Z.); 15865619762@163.com (J.Z.);

**Keywords:** gold tailings, porous ceramics, foaming mechanism, thermal insulation, heat resistance

## Abstract

This study demonstrates a high-value pathway for fabricating porous ceramics by utilizing gold tailings (GT) as the principal raw material, with silicon carbide (SiC) as a high-temperature foaming agent. The microstructure, mechanical strength, and thermal conductivity were tailored by adjusting GT content, sintering temperature, raw material particle size, and foaming agent dosage. The optimized ceramics exhibit a total porosity of 60.1–83.7%, a compressive strength of 3.25–7.18 MPa, and a thermal conductivity of 0.15–0.32 W·m^−1^·K^−1^. These properties not only meet, but in fact exceed the key requirements specified in the Chinese National Standard GB/T 16533-1996 for porous thermal insulation ceramics. Notably, the materials achieve an optimal balance between high porosity and adequate mechanical strength. The findings confirm that gold tailings can be effectively valorized to produce standardized, porous ceramics suitable for industrial thermal insulation applications.

## 1. Introduction

The intensive and long-term exploitation of gold resources in China, the world’s largest gold producer, has resulted in the accumulation of vast quantities of gold mine tailings (GT). Statistics indicate an annual generation of 200 million tons of GT, with a historical stockpile exceeding 1.5 billion tons. However, the comprehensive utilization rate remains below 20% [1]. These stockpiles not only occupy extensive land resources, but also pose significant environmental and safety hazards, including risks of soil contamination, water eutrophication, and heavy metal bioaccumulation [2]. With increasingly stringent environmental regulations and restrictions on new tailings pond construction, the resource utilization of GT has become a critical challenge for the sustainable development of the mining industry.

Current disposal methods for GT, such as underground backfilling, land reclamation, and the production of building materials, are often hampered by low economic return, high processing costs, or insufficient product durability. While methods for recovering residual valuable metals (e.g., Au, Ag) exist, they are typically cost-intensive and cannot consume the bulk of the tailings volume [3]. Consequently, developing high-value-added utilization pathways is imperative.

In this context, the synthesis of functional materials like ceramsite, microcrystalline glass, and particularly porous ceramics from GT has emerged as a promising direction. The chemical composition of GT, rich in quartz, feldspar, and mica, provides a suitable raw material base for fabricating silicate-based porous ceramics. Porous ceramics are a class of materials characterized by a cellular structure, endowing them with low density, high specific surface area, excellent thermal stability, and notable insulation properties [4]. These attributes make them highly suitable for diverse applications, including structural components [5], filtration/adsorption media [6], thermal insulators [7], and catalyst supports [8].

Various techniques have been developed to fabricate porous ceramics, including chemical foaming [9], sacrificial template methods [10], pore-forming agent addition [11], 3D printing [12], and foam-gel casting [13]. Among these, the high-temperature foaming method using a foaming agent stands out for its simple process, stable and uniform pore formation, and suitability for industrial-scale production, making it ideal for preparing closed-cell porous ceramics [14]. Silicon carbide (SiC) is a widely used and cost-effective foaming agent. It functions by oxidizing at high temperatures (e.g., around 980 °C) within a molten ceramic matrix to generate gas bubbles [15]. Studies have confirmed that the oxidation of SiC in an alkaline melt during firing is a key mechanism for pore formation in systems like porcelain polishing residue [16,17].

These studies indicate that controlling the sintering process allows for high-temperature foaming agents to generate gas within inorganic matrices, such as silica-rich tailings, to create porous structures. However, research on the preparation of porous ceramics specifically from GT remains limited. For instance, Wang et al. [18] prepared porous ceramics from a mixture of alkali slag and GT, achieving a bulk density of 0.5 g/cm^3^ and a compressive strength of 0.62 MPa. In another study, Uribe et al. [19] utilized gold silver tailings with Na_2_CO_3_ as a foaming agent, producing foam ceramics with an average pore size of 0.80 mm, a density of 0.7–0.8 g/cm^3^, and a compressive strength of 0.4–0.6 MPa.

While these studies demonstrate feasibility, the reported mechanical strengths are relatively low, limiting potential applications. Therefore, a significant research gap exists in developing high-performance porous ceramics from GT. Building on previous work, this study aims to fabricate GT-based porous ceramics with superior properties by systematically optimizing the foaming agent (type and dosage) and sintering process conditions. The focus will be on understanding the relationship between the resulting microstructure and key functional properties, such as thermal conductivity and mechanical strength, to advance the high-value utilization of this industrial waste.

## 2. Materials and Methods

### 2.1. Materials and Experiment Process

The solid raw material GT used in this experiment was provided by a mining area in Zhaoyuan, China. PL and GP are provided by a mining company in Zibo, solvent used is deionized water. Boric acid, as a foam stabilizer, was provided by a chemical company in Zibo. This study employed two industrial-grade SiC blowing agents, designated SiC1# and SiC2#, which were supplied by a chemical plant in Zibo. Their median particle sizes (D_50_) were 28.7 μm and 3.0 μm, respectively.

### 2.2. Sample Preparation

**Figure 1:**
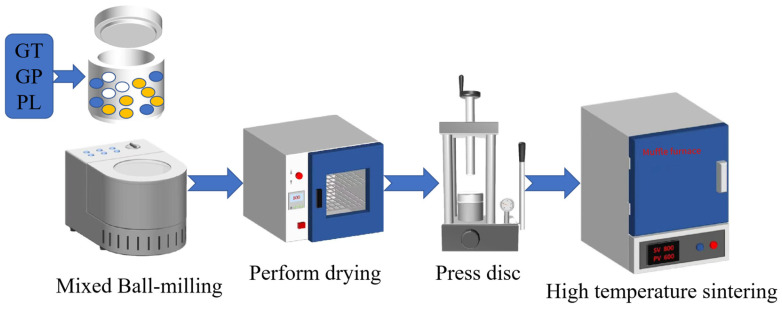
Preparation process flowchart of porous ceramics.

All raw materials were initially dried at 100 °C for 2 h. According to the formulations listed in Table 1, gold tailings and auxiliary materials (PL, GP, and SiC) were precisely weighed and mixed in a high-speed ball mill (JJ-5, Hebei Yaxing Highway Construction Instrument Factory, Cangzhou, China) operated at 1500 r/min for 2 h to obtain a homogeneous powder mixture. Subsequently, the mixed powder was uniaxially pressed at 15 MPa to form disc-shaped green compacts with dimensions of Φ50 × 10 mm. For the foaming samples (with SiC), the compacts were sintered in a muffle furnace (SX-G80133, Tianjin Zhonghuan Electric Furnace Co., Ltd., Tianjin, China) at 1050–1080 °C (heating rate: 3 °C/min; dwell time: 60 min). After natural cooling, the resulting foamed bodies were cut into 25 mm cubes for compressive testing. In contrast, the dense reference samples (formulations A1–A4 without SiC) were sintered at 1050 °C and then sectioned into rectangular specimens measuring 40 mm × 80 mm for characterization. The entire preparation process is detailed in the flowchart presented in Figure 1.

### 2.3. Testing and Characterization

The phase composition of the samples was tested using the D8-02 (Bruker, Karlsruhe, Germany) X-ray diffractometer with a Cu target. The chemical composition of the raw materials was detected using the ZSX100e X-ray fluorescence spectrometer (Rigaku, Osaka, Japan). The particle size distribution of the raw materials was tested using the Hydro 2000SM(A) laser particle size analyzer (Malvern Panalytical, Malvern, UK). Based on Archimedes’ principle, a static balance with a precision of 0.01 mg was used to determine the porosity, bulk density and true porosity of the foamed porous ceramics. The microstructure of the porous ceramic materials was observed using a Quanta 250 (Thermo Fisher Scientific, Waltham, MA, USA); electron scanning microscope, and the pore size distribution of the measured sample was statistically analyzed using Nano-measure software (version 1.2). The compressive strength of the porous ceramics cubic samples (25 mm × 25 mm × 25 mm) was measured using an Instron-5969 (Instron, Norwood, MA, USA) universal testing machine at a constant crosshead speed of 0.5 mm/min. The micro-area composition of the sample surface was detected using Inca X-ray energy spectrometers (Shanghai, China). The thermal conductivity of porous ceramics at room temperature was measured using the TC-3200 (Xi’an Xiaxi Electronic Technology Co., Ltd., Xi’an, China) flat plate thermal conductivity meter. The room-temperature thermal conductivity of the dense ceramic matrix, prepared from gold tailings with varying compositions, was measured by laser flash analysis (LFA) using a LFA-427 instrument (Netzsch Instrument Manufacturing Co., Ltd., Selby, Germany). The VL2000DX-SVF18SP (Micang production office of Japan Co., Ltd., Kotoku, Tokyo, Japan.) high-temperature confocal laser microscope was used to dynamically observe the high-temperature melt in foaming experiments, achieving in situ observation of bubble growth during high-temperature sintering and observing the microstructure and dynamic changes in pores. The reported mechanical and thermal properties, including the compressive strength (tested on at least five independent samples) and thermal conductivity (n ≥ 3), are presented as the mean value ± standard deviation.

## 3. Results and Discussion

### 3.1. Characterization of Raw Materials

#### Mineralogical and Chemical Characterization of Raw Materials

**Figure 2:**
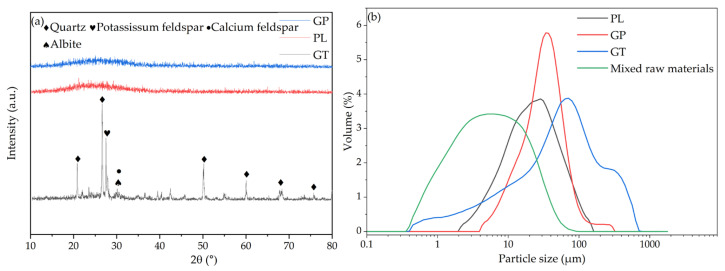
XRD and particle size; (**a**) XRD; (**b**) particle size.

Based on the characterization results, the major oxide compositions of the raw materials are summarized in Table 2. The gold tailings (GT) are primarily composed of SiO_2_ and Al_2_O_3_, with quartz (SiO_2_) and potassium feldspar (KAlSi_3_O_8_) identified as the main crystalline phases. Particle size analysis, as illustrated in Figure 2, reveals distinct initial particle sizes for the raw materials: GT has a D_50_ of 58 μm, while the as-received perlite (PL) and glass powder (GP) are considerably finer, with D_50_ values of 20.91 μm and 30.48 μm, respectively. This notable difference in initial particle size could potentially lead to segregation and affect mixing homogeneity. To mitigate this and enhance the reactivity of the mixture, the raw materials were jointly ball-milled for two hours. This process successfully resulted in a homogeneous mixture with a significantly reduced D_50_ of 5.60 μm.

### 3.2. Pore Size and Bulk Density

**Figure 3:**
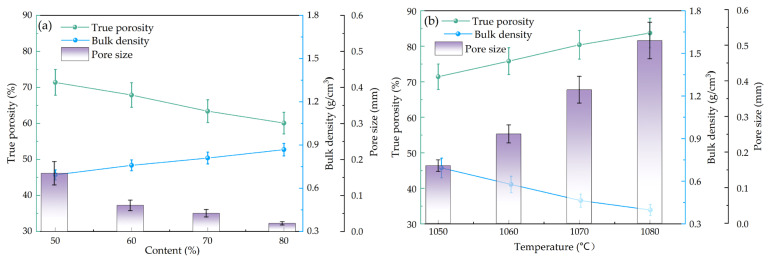
Bulk density, true porosity, and pore size fired at 1050 °C with different gold tailings amounts and bulk densities; and true porosity and pore size of samples fired at 1050–1080 °C; (**a**) Different components of gold tailings; (**b**) Different sintering temperatures.

The composition, particle size and sintering temperature of raw materials will affect the true porosity, bulk density and pore size of porous ceramics. Figure 3 shows the effect of the composition of different gold tailings and sintering temperature on porous ceramic materials. As shown in Figure 3, with the increase of gold tailings amount, the true porosity gradually decreased from 71.4% to 60.1%, and the bulk density increased from 0.693 to 0.868 g/cm^3^. The data is similar to the true porosity of porous ceramics prepared by Huang using tailings [20]. And the pore size was decreased significantly from 0.162 mm to 0.023 mm. Analyzing the reasons from the perspective of raw materials, during sintering, the main components such as SiO_2_ and Al_2_O_3_ in gold tailings could promote the chemical bonding between particles and the formation of new crystalline phases, resulting in liquid-phase connections or crystalline phase leads to the more compact structure, thereby reducing porosity and pore size. When the composition of gold tailings is determined to be 50% (A1), the data of porous ceramic samples sintered at 1050–1080 °C indicate that true porosity and pore size increase slightly with sintering maximum true porosity 83.7% and pore size 0.0513 mm at the sintering temperature of 1080 °C however the bulk density reached its lowest point at 1080 °C this tend suggests that higher sintering temperature lead to the significant increase in pore size affects the decrease in bulk density and looser structure of porous ceramics.

Ye proposed that the particle size of raw powder also affected the porosity and pore size of porous ceramics [21]. The particle size of raw materials also affects the microstructure of the porous ceramics. Based on the proportions of raw materials in the A1 sample, the gold tailings, replaced with untreated raw gold tailings and gold tailings milled by dry ball milling for 120 min, while the silicon carbide foaming agents were as well as replaced with different particle sizes, named SiC1# and SiC2#. The other raw materials remained the same, and the new samples were numbered B1, B2, B3, and B4, as detailed in Table 3. After mixing all raw materials together, mix and ball mill for 20min to make them mixed evenly. The foaming temperature of the new samples was 1050 °C with a holding time of 1 h.

**Figure 4:**
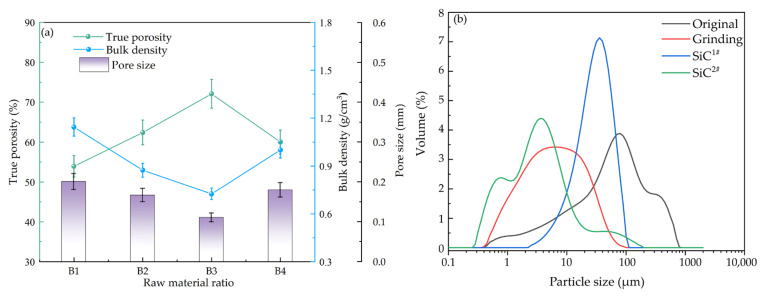
Bulk density, true porosity, and pore size diagrams of porous ceramics with different particle sizes of raw materials, and particle size distribution diagram of raw materials; (**a**) Bulk density, true porosity, and pore size diagrams; (**b**) particle size distribution diagram.

The aforementioned results have identified the formulation with 50 wt.% gold tailings (GT) sintered at 1050 °C (Sample A1) as a promising base composition. To further optimize the microstructure, specifically to achieve higher porosity and a finer, more uniform pore structure, we investigated the effect of raw material particle size on this optimized base formulation. The GT and silicon carbide (SiC) foaming agent were selected as the key variables for this subsequent stage of process refinement. The particle size distribution of each raw material was measured using a Hydro-2000SM (A) laser particle size analyzer (Malvern Panalytical, UK), and the results were presented in Figure 4 The particle size distribution of the untreated gold tailings was relatively wide, ranging from 0.5 μm to 1000 μm, with a D50 of approximately 58 μm. Following 20 min of ball milling, the particle size distribution of the gold tailings narrowed significantly, ranging from 0.5 μm to 100 μm, with a D50 of 5.6 μm. This ball milling treatment effectively refined the particles and concentrated their size distribution. The particle size distribution characteristics of the two silicon carbide foaming agents differed. Specifically, SiC1# exhibited a relatively narrow particle size distribution range but larger particle sizes, with a D50 of approximately 28.7 μm, while SiC2# had a broader particle size distribution range but smaller particle sizes, with a D50 of approximately 3.0 μm.

Figure 4 showed the Bulk density, true porosity, and pore size diagrams of porous ceramics with different raw particle sizes after foaming at 1050 °C. It was found that the pore morphology and distribution of the samples prepared from ball-milled gold tailings were finer and more uniform than those of the unball-milled samples. The smaller the powder particle size, the larger the specific surface area and the higher the surface energy. Under the same temperature, the smaller particles with higher surface energy were more easily melted or participated in reactions to form a liquid phase, providing more favorable liquid phase conditions for foaming and thus resulting in more ideal pore size and distribution. This effect could also be observed from the test results of the samples’ bulk density, true porosity, and pore size, as shown in Figure 4. When using a foaming agent with a D50 of 28.7 μm, as the particle size of gold tailings decreased from 58 μm to 5.6 μm, the true porosity of the porous ceramic increased from 54% to 67%, the bulk density decreased from 1.15 g/cm^3^ to 0.85 g/cm^3^, and the average pore size decreased from 0.2 mm to 0.16 mm. As shown in Figure 4, the porous ceramic prepared using SiC with a D50 of 3.0 μm as the foaming agent exhibited superior pore morphology and distribution compared to the sample with a D50 of 28.7 μm. When the particle size of gold tailings was 5.6 μm, the porous ceramic sample prepared demonstrated the true porosity of 74%, The bulk density of 0.63 g/cm^3^, and the average pore diameter of approximately 0.11 mm. the performance of Sample B4 (coarse GT + fine SiC2#) provides key insights into the synergistic effect of raw material particle sizes. With a true porosity of 60%, a bulk density of 1.0 g/cm^3^, and an average pore size of 0.18 mm, its properties are intermediate to those of B1 and B3. This confirms that while a fine foaming agent is critical for generating numerous small pores, the matrix particle size concurrently governs the stability and growth of these pores. The coarser GT matrix in B4 appears to offer less resistance to pore coalescence during foaming compared to the finer B3 matrix, resulting in larger pores and lower overall porosity from the perspective of the foaming mechanism, each SiC particle could be regarded as a gas nucleation-bubble growth unit. Under the same foaming temperature and time, the size of SiC particles determines the size of the formed bubbles: the smaller the SiC particles, the smaller the pore dimensions. Additionally, when the same weight of SiC was added to the green body, smaller SiC particles resulted in a higher number of particles, a larger specific surface area, and a larger contact area with oxygen. According to chemical reaction kinetics, a larger interfacial contact area can accelerate solid-state reactions, which in the context of composite materials often leads to the in situ generation of gaseous by-products or volatile phases at high temperatures, thereby increasing porosity. This phenomenon is consistently observed in various thermoelectric composites, where the incorporation of secondary phases significantly alters the microstructure. For instance, in Ni_0.05_Mo_3_Sb_5.4_Te_1.6_/SiC composites consolidated by hot-pressing, the addition of SiC nanoparticles resulted in an 80% increase in surface area and a remarkable 280% increase in cumulative pore volume, which was directly correlated with a decrease in bulk density [22]. A similar trend, where the introduction of a second phase leads to compromised densification, has also been reported in other composite systems, such as Al_2_O_3_/Lead telluride and NiSb/Lead telluride [22,23]. Consequently, this reaction-induced pore generation effectively increases the true porosity of the material and results in a lower bulk density.

Notably, although sample B3 (with milled tailings and fine SiC) exhibited a more uniform and finer pore microstructure, its macroscopic mechanical and thermal properties were not superior to those of sample A1 (with raw tailings and coarse SiC). This phenomenon indicates that the optimization of the microstructure does not always translate linearly into an improvement in macroscopic properties. Potential reasons include the following: the higher porosity in sample B3, while beneficial for thermal insulation, also weakened the load-bearing capacity of the material. Furthermore, the excessively fine pore structure may lead to thinner pore walls, which are more susceptible to stress concentration and failure under load. In contrast, sample A1, although slightly less uniform in pore structure, possessed thicker pore walls and a more continuous structure, thereby achieving a better balance between compressive strength and thermal conductivity.

### 3.3. SEM and Pore Size Distribution

**Figure 5:**
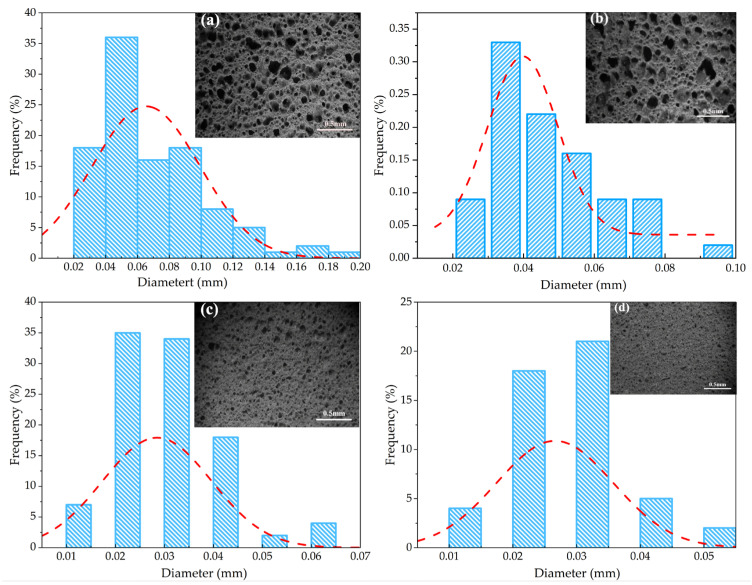
Morphology and pore size distribution of porous ceramics with different gold tailings amounts; (**a**) 50%; (**b**) 60% (**c**) 70% (**d**) 80%; The blue hatched histograms correspond to the pore size distribution, the red dashed curves represent the Gaussian fitting results (Same below).

Figure 5 presents the microstructure images and pore size distribution of the porous ceramic samples. As illustrated in the figure, with the increase in GT content, the pore size of the samples decreased significantly, from 0.162 mm to 0.023 mm. Moreover, the Gaussian fitting results of the pore size distribution exhibit obvious differences among samples with varying GT contents this directly indicates that GT content exerts a prominent regulatory effect on the pore size of the porous ceramics, and the optimal GT content was determined to be 50%. From the perspective of raw material composition and sintering mechanism, the key components SiO_2_ and Al_2_O_3_ in gold tailings play a critical role in modifying the ceramic structure during sintering. Specifically, SiO_2_ and Al_2_O_3_ can form a low-melting-point eutectic phase at the sintering temperature, which not only promotes the chemical bonding between ceramic matrix particles but also induces the nucleation and growth of new crystalline phases aluminosilicate phases. This dual effect liquid-phase bridging + crystalline-phase bonding enhances the connectivity between particles, driving the ceramic structure toward densification ultimately manifesting as reduced porosity and smaller pore size.

The reduction in porosity and pore size can be attributed to the synergistic effect of the raw material composition and the sintering process. From a materials perspective, the main components in gold tailings, such as SiO_2_ and Al_2_O_3_, can act as fluxing agents or form secondary crystalline phases during sintering, promoting chemical bonding between particles and leading to a more compact structure through liquid-phase or crystalline-phase connections. Critically, the efficiency of this densification process is highly dependent on the consolidation method. This is clearly demonstrated in thermoelectric composites, where the same matrix material consolidated with different methods and additives results in vastly different densities. For instance, in a Ni_0.05_Mo_3_Sb_5.4_Te_1.6_ system, composites with Al_2_O_3_ nanoparticles consolidated via spark plasma sintering (SPS) achieved a high relative density of up to 98.5%, whereas analogous composites with SiC nanoparticles consolidated by conventional hot-pressing reached a maximum relative density of only 94.2% [22]. The intense Joule heating, pressure, and rapid mass transfer in SPS are known to be far more effective in eliminating pores and achieving superior densification compared to hot-pressing [22]. Therefore, the observed densification in our samples is not only a function of the chemical activity of the gold tailings components but also a direct consequence of the effective sintering process that facilitates the particle bonding they promote.

**Figure 6:**
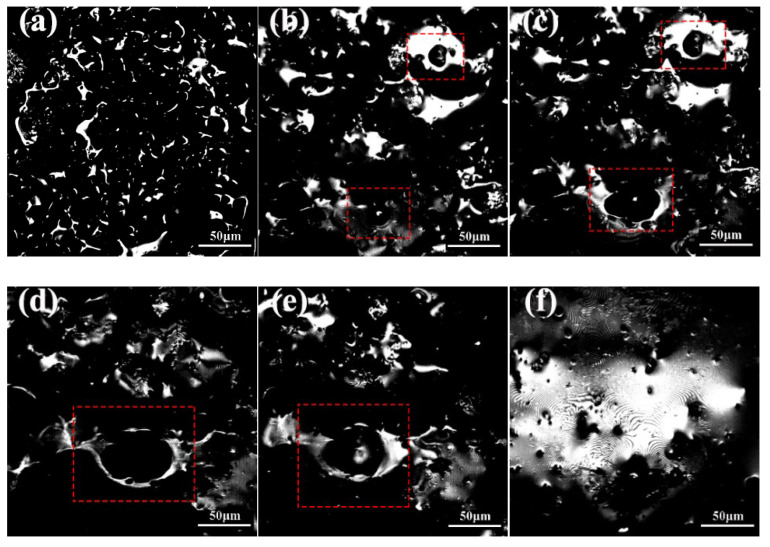
Confocal laser microscopy images of porous ceramics: (**a**) 1000 °C; (**b**) 1050 °C; (**c**) 1060 °C; (**d**) 1070 °C; (**e**) 1080 °C; and (**f**) 1090 °C.

To investigate the effect of temperature on the pore formation process, the experiment selected the samples with 50% gold tailings (A1) as the research objects. High-temperature laser confocal microscopy (CLSM) dynamic monitoring technology was used to observe the entire foaming process under high temperature, and the foaming state photographs were captured at 1000 °C, 1050 °C, 1060 °C, 1070 °C, 1080 °C, and 1090 °C, as shown in Figure 6.

As the temperature continued to rise to 1050 °C, potassium and Na_2_O reacted with the SiO_2_ layer, forming a low-viscosity, flowing silicate phase, which destroyed the passivation layer. This process eliminated the restriction on oxygen transfer, promoting the silicon carbide oxidation reaction into a self-accelerating stage. The gaseous products, primarily CO, were increased rapidly, leading to a significant growth in gas volume. Sub-micron spherical pores began to appear in the melt. At this point, the high viscosity of the melt caused bubbles to grow slowly, preserving a relatively thick pore wall structure between adjacent pores.

Upon further increasing the temperature to 1060 °C, the rheological properties of the melt underwent significant changes. The system’s viscosity began to decrease, and bubble expansion and growth became easier. During this process, bubbles subjected to shear stress form ellipsoidal/pear-shaped morphologies, and the interstitial membrane thickness decreased. When the temperature surpassed the critical point of 1070 °C, the pores entered the confluent stage. Adjacent bubbles merged through the membrane perforation mechanism, forming polyhedral pore structures with shared interfaces. At this point, the system surface can achieve a new equilibrium state through pore wall reconstruction.

When the temperature reaches 1080 °C (Figure 6e), the balance between melt tension and internal bubble pressure reversed. The equilibrium maintained by the melt surface tension was disrupted, which led to bubble rupture. In the final stage (Figure 6f), at 1090 °C, the porosity structure completely disappeared, Gold tailings based porous ceramics undergoes severe melting at 1090 °C: Viscosity plummets:Melt viscosity is critical for sustaining bubble structure higher viscosity “traps” bubbles. At 1090 °C, low viscosity fails to restrain bubble growth/merging. The decrease in surface tension weakens the melt’s ability to maintain discrete bubble interfaces, making bubble rupture and coalescence easier. Thus, even without excessive gas generation, the melt’s physical stability, governed by viscosity and surface tension, collapses at 1090 °C, further promoting pore interconnection. This confirms that the entire decomposition reaction of silicon carbide has been completed and that the foaming process has reached its terminal state with no further gas generation.

**Figure 7:**
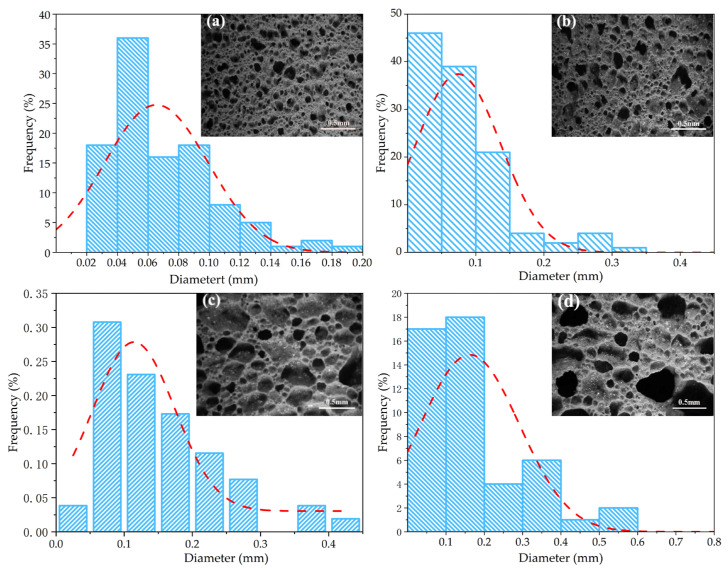
Morphology and pore size distribution of porous ceramics sintered at different temperatures: (**a**) 1050 °C; (**b**) 1060 °C; (**c**) 1070 °C; and (**d**) 1080 °C.

Based on the observation of the dynamic foaming process using confocal microscopy, the suitable foaming temperature range for porous ceramics made from gold tailings is 1050–1080 °C. To further optimize the process parameters, the samples with 50% gold tailings were subjected to foaming sintering at temperatures of 1050 °C, 1060 °C, 1070 °C, and 1080 °C, respectively, and were labeled as A1, A5, A6, and A7 in Table 1. The aim was to investigate the effect of temperature on the properties of porous materials. Figure 7 illustrates the morphological characteristics of porous ceramic samples prepared at various sintering temperatures. The temperature above 1070 °C has little effect on the results of Gaussian fitting. At 1050 °C, the pores are uniform and have a relatively regular shape. As the temperature gradually increases, irregular large pores are formed, and there is a phenomenon of interconnection between the pores. Figure 7 shows the temperature above 1070 °C has little effect on the results of Gaussian fitting. At 1050 °C, the pores are uniform and have a relatively regular shape. As the temperature gradually increases, irregular large pores are formed, and there is a phenomenon of interconnection between the pores.

**Figure 8:**
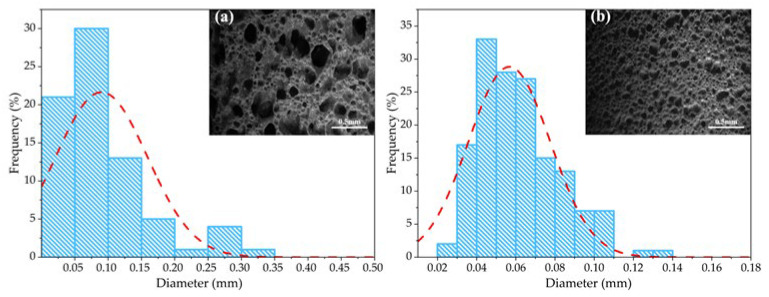
Morphology and pore size distribution of porous ceramics prepared from different gold tailings particle sizes; (**a**): D50 5.6 μm; (**b**): D50 58 μm.

**Figure 9:**
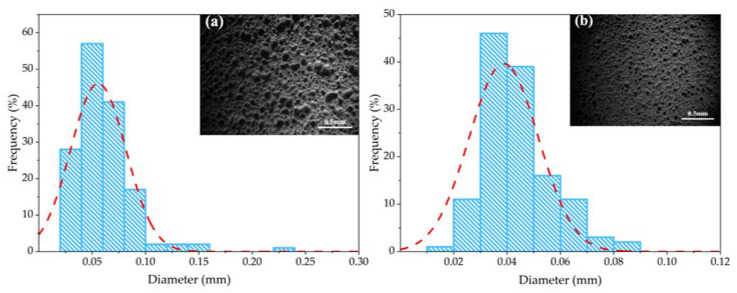
Morphology and pore size distribution of porous ceramics prepared with different silicon carbide particle sizes; (**a**) D50 3.0 μm; (**b**) D50 28.7 μm.

As shown in Figure 8 and Figure 9, the porous ceramic prepared using SiC with a D50 of 3.0 μm as the foaming agent exhibited superior pore morphology and distribution compared to the sample with a D50 of 28.7 μm. The aperture size is more even, and the distribution is more uniform. When the particle size of gold tailings was 5.6 μm, the porous ceramic sample prepared demonstrated the true porosity of 74%, the bulk density of 0.63 g/cm^3^, and the average pore diameter of approximately 0.11 mm. From the perspective of the foaming mechanism, each SiC particle could be regarded as a gas nucleation-bubble growth unit. Under the same foaming temperature and time, the size of SiC particles determines the size of the formed bubbles: the smaller the SiC particles, the smaller the pore dimensions. Additionally, when the same weight of SiC was added to the green body, smaller SiC particles resulted in a higher number of particles, a larger specific surface area, and a larger contact area with oxygen. According to chemical reaction kinetics, a larger contact area can accelerate the reaction rate, thereby promoting the generation of more gaseous products at high temperatures. This mechanism leads to an increase in the true porosity of the porous ceramic material while decreasing its bulk density.

Such a correlation between contact area, reaction kinetics, and structural evolution has been widely observed in various composite systems, including Al_2_O_3_/lead telluride, SiC/lead telluride, and NiSb/lead telluride composites. For instance, Ref. [22] reported that in SiC-based composites, the enhanced interfacial contact area between SiC and the matrix phase accelerated oxidation or interfacial reaction kinetics, which in turn regulated pore formation and density. Additionally, Ref. [22] illustrated that in ceramic/thermoelectric composites Al_2_O_3_/lead telluride, a larger contact area facilitated mass transfer and reaction efficiency during sintering, ultimately influencing the composite’s porosity and bulk density in a manner consistent with kinetic principles. The consistency between our findings and these studies further validates the reliability of the contact area-dependent reaction kinetics mechanism in governing the porous ceramic’s structure and properties. 

This study used Sample A1 as a reference and set the amount of silicon carbide foaming agent to 0.0 wt.%, 0.10 wt.%, 0.20 wt.%, 0.30 wt.%, 0.50 wt.%, and 1.00 wt.% to obtain new sample groups, with new sample numbers C1–C5 as detailed in Table 1. The foaming temperature was maintained at 1050 °C for 1 h. By analyzing the microstructure, bulk density, and true porosity of the samples, the influence and mechanism of the silicon carbide foaming agent dosage were investigated.

**Figure 10:**
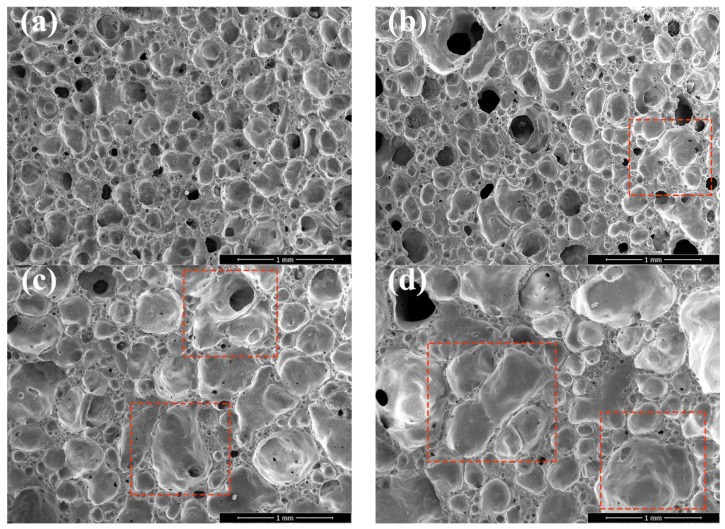
The SEM images of sintered samples with different SiC contents: (**a**) 0.10 wt.%; (**b**) 0.20 wt.%; (**c**) 0.30 wt.%; and (**d**) 0.50 wt.%.

Figure 10 presents SEM images of sintered porous ceramic samples with varying SiC contents, where (a), (b), (c), and (d) correspond to SiC contents of 0.10 wt.%, 0.20 wt.%, 0.30 wt.%, and 0.50 wt.%, respectively. Red boxes in the images highlight typical regions of pore merging or morphological evolution for clearer observation. SiC undergoes oxidation at high temperatures by the reaction such as: SiC + O_2_ → SiO_2_ + CO/CO_2_ to generate gaseous products. With increasing SiC content, the volume of generated gas also increases, which further promotes bubble growth and enhances mutual bubble merging during sintering thus inducing significant changes in pore structure, porosity, and bulk density. When SiC content was lower than 0.10 wt.% in Figure 10a, the generated gas was insufficient to support full pore growth, resulting in smaller pore sizes and relatively higher bulk density. As SiC content increased 0.20 wt.% in Figure 10b, 0.30 wt.% in Figure 10c, and 0.50 wt.% in Figure 10d, more gas elevated internal bubble pressure. When this pressure exceeded the surface tension of the ceramic matrix’s molten phase, bubbles ruptured and merged with adjacent bubbles highlighted by red boxes in Figure 10b–d. Simultaneously, some bubbles migrated to the surface, ruptured, and formed open pores causing a notable decrease in pore uniformity most evident in Figure 10d with the highest SiC content.

### 3.4. Strength and Thermal Conductivity

**Figure 11:**
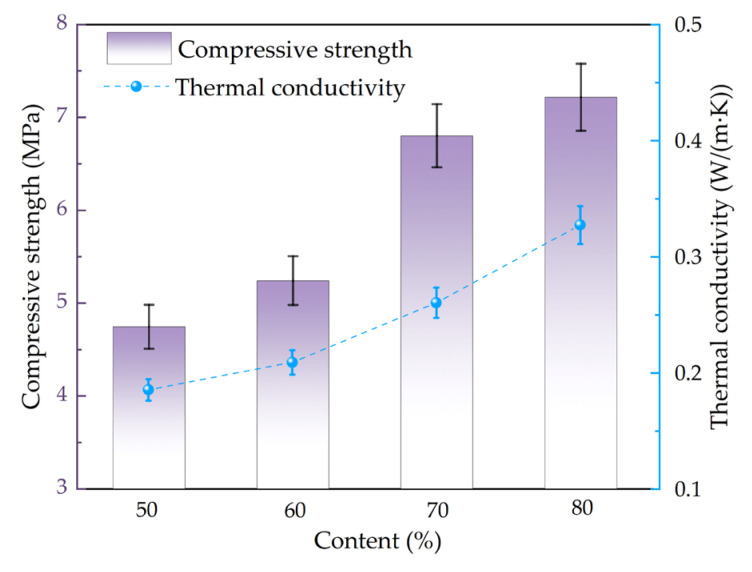
Compressive strength and thermal conductivity of porous ceramic materials fired at 1050 °C with different gold tailings amounts.

As shown in Figure 11, with the increase in the amount of gold tailings, the compressive strength of porous ceramics increased from 4.62 MPa to 7.18 MPa, and the thermal conductivity increased from 0.186 W/(m·K) to 0.32 W/(m·K). The enhancement of the compressive strength came from the reduced pore size and porosity of the materials with the increase in the amount of gold tailings as shown in Figure 5 and Figure 11 The increase in material density strengthens the continuity of phonon conduction pathways, thereby improving the thermal conductivity.

This can be explained by the parallel thermal conduction model of porous ceramics, as shown in Equation (1).

λ = V_1_λ_1_ + V_2_λ_2_ (1)

where λ_1_ is the thermal conductivity of the dense ceramic matrix, λ_2_ is the thermal conductivity of air, V_1_ is the volume fraction of the dense ceramic matrix, and V_2_ is the volume fraction of the pores.

**Figure 12:**
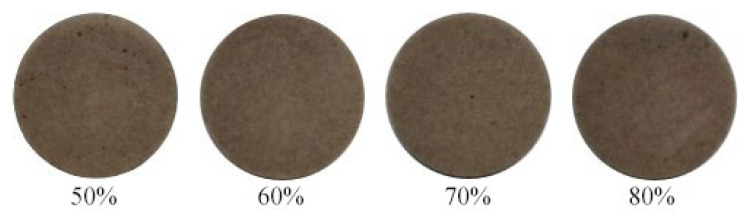
Compact bodies with different tailings amounts.

In this study, a laser thermal conductivity instrument was used to measure the thermal conductivity of dense ceramic samples containing 50% to 80% gold tailings at room temperature. The dense ceramic samples made from gold tailings are shown in Figure 12, and the results of the thermal conductivity measurements are presented in Table 4.

As shown in Table 4, increasing the gold tailings content from 50% to 80% led to a systematic increase in the bulk density of the dense samples from 2.06 g/cm^3^ to 2.24 g/cm^3^, accompanied by a decrease in porosity from 71.4% to 60.1%. This densification process enhanced the crystalline phase components, which formed a continuous solid-phase framework, while the liquid phase filled the internal pores, ultimately resulting in a denser structure. Consequently, the thermal conductivity increased from 0.186 W/(m·K) to 0.327 W/(m·K). This enhancement can be attributed to the reduced porosity, which minimizes phonon scattering sites and establishes more continuous pathways for phonon quantitatively decouple the effect of porosity from the intrinsic material properties on thermal conductivity, the Maxwell-Eucken equation was employed, following the methodology detailed in Nandihalli et al. for nanocomposite thermoelectric materials [22].

Concomitantly, the thermal conductivity increased from 1.26 W/(m·K) to 1.53 W/(m·K). This enhancement can be fundamentally understood through the lens of phonon transport. The increased density directly reduced porosity, which in turn minimized phonon scattering sites and allowed for more continuous solid-phase contact. This creates a greater number of efficient pathways for phonon propagation, thereby enhancing the material’s thermal conductivity [24]. This observation is consistent with the established understanding that the lattice thermal conductivity is highly dependent on the material’s density and microstructure, as a denser structure with fewer imperfections facilitates more effective phonon travel [24]. Therefore, the decrease in porosity with higher gold tailings content directly resulted in an increased overall thermal conductivity of the material. The equation is a Maxwell Eucken equation:

(2) $$k_{p}=\;k_{0}\times \frac{1-p}{1+\beta_{p}}$$

where k_p_ is the measured thermal conductivity, k_0_ is the pore-corrected thermal conductivity, p is the porosity and β is an empirical factor dependent on pore morphology (typically between 1 and 3 for spherical pores) [22]. Applying this correction to our data reinforces the conclusion that the observed rise in thermal conductivity is primarily due to the reduction in porosity, which facilitates more efficient phonon transport by reducing scattering centers. Conversely, when investigating the effect of sintering temperature (1050–1080 °C) on porous ceramics, an inverse trend was observed. The increase in sintering temperature resulted in a rise in porosity from 71.4% to 83.7%, which in turn caused the thermal conductivity to decrease from 0.186 W/(m·K) to 0.150 W/(m·K). This phenomenon is consistent with the principle that increased porosity introduces more phonon-scattering interfaces, thereby impeding thermal transport. The analysis confirms that the thermal conductivity of these porous ceramics is predominantly governed by their porosity, and the Maxwell-Eucken model provides a robust theoretical framework to correlate microstructural evolution with macroscopic thermal properties.

**Figure 13:**
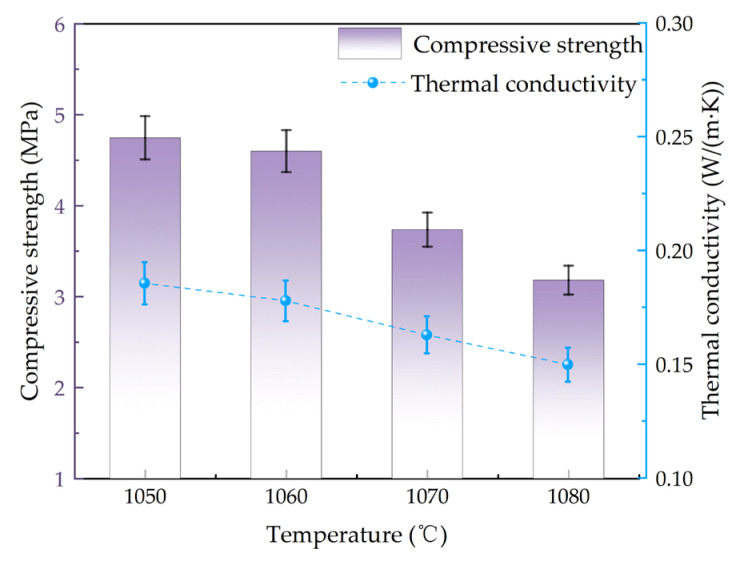
Compressive strength and thermal conductivity of porous ceramics at different sintering temperatures.

The strength and thermal conductivity of the aforementioned samples were tested as shown in Figure 13. As the temperature increased from 1050 °C to 1080 °C, the compressive strength of the material was decreased from 4.75 MPa to 3.25 MPa with the decrease of approximately 32%. The thermal conductivity was decreased from 0.18 W/(m·K) to 0.15 W/(m·K). The compressive strength meets the requirements of GB/T 16533-1996 [25] for porous ceramics [25]. Both strength and thermal conductivity decreased with increasing sintering temperature, essentially due to the influence of pore structure on these two properties. The compressive strength of porous ceramics was primarily influenced by two factors. The first factor was the effect of pore size and distribution characteristics. As shown in Figure 3, with increasing temperature, the porosity of the material increased, pore size gradually became larger, and the distribution uniformity decreased. The increase in porosity reduced the effective load-bearing area of the solid matrix material. When the material is subjected to external loading, stress concentration tended to occur around the pores, which reduced the limit load of the material.

### 3.5. XRD

**Figure 14:**
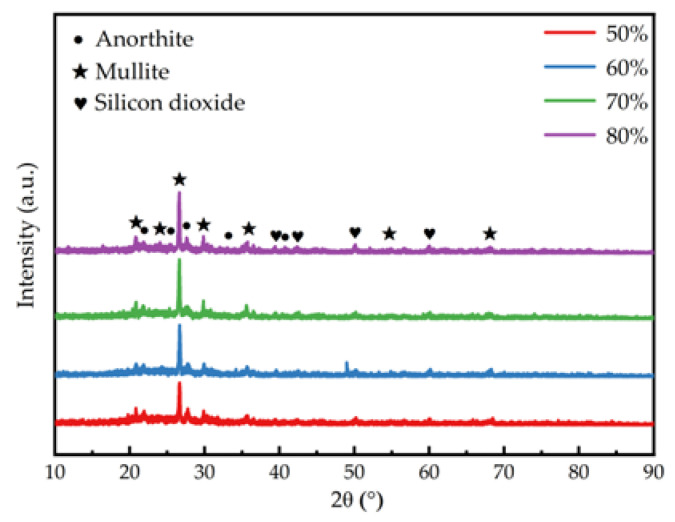
XRD patterns of porous ceramic materials fired at 1050 °C with different gold tailings amounts.

Figure 14 shows the phase compositions of different components contains anorthite mullite silicon dioxide. From the perspective of the generated crystalline phases, as the increases of gold tailings addition. In the sintering process, the oxides in gold tailings first melt to form a liquid phase under high temperature, which serves as a medium for mass transfer and promotes the diffusion and rearrangement of particles. With the progress of sintering, the components in the liquid phase gradually crystallize into quartz and mullite under the driving force of phase equilibrium. As the addition of gold tailings increases, more crystalline phases are generated, which continuously consume the components in the liquid phase, resulting in a reduction in the residual liquid phase The increase in crystalline phases enhances the bonding strength between particles during sintering, as the rigid crystalline frameworks form a more stable load-bearing structure, restricting the expansion space of pores. Meanwhile, the reduction in the residual liquid phase increases the overall viscosity of the system, which enhances the resistance to bubble growth during foaming. These two effects together result in increased material densification, decreased porosity, and reduced pore size. This outcome is illustrated in Figure 4.

**Figure 15:**
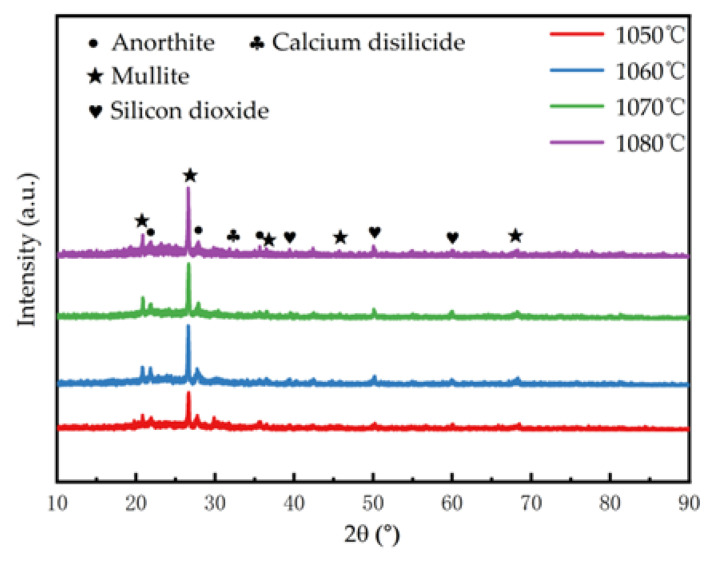
XRD patterns of porous ceramic materials fired at different temperatures.

Figure 15 shows that matrix of porous ceramic materials was a mixture of crystalline phases and glass phases enriched in silicon and aluminum. As the temperature rose, the tendency for silicon-aluminum phases to undergo eutectic reactions increased, leading to the melting of some crystalline phases or the formation of glassy melts. The reduction in crystalline phases and the increase in glass phases weakened the overall strength of the solid matrix material.

### 3.6. Mechanism Analysis

**Figure 16:**
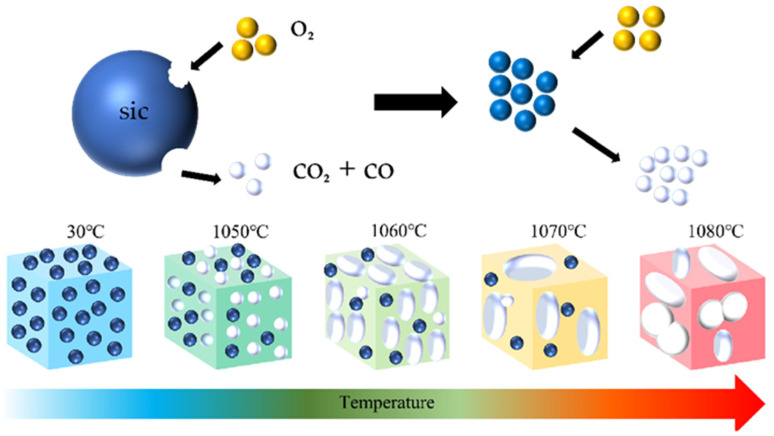
Schematic diagram of the reaction mechanism of silicon carbide and the process of pore generation and growth.

During the initial stage of sintering at 1000 °C, a dense SiO_2_ passivation layer rapidly formed on the surface of silicon carbide. This passivation layer inhibited the diffusion and penetration of oxygen molecules into the interior of the particles and prevents the sustained occurrence of the reactions SiC + 2O_2_ → SiO_2_ + CO_2_ or SiC + O_2_ → SiO_2_ + CO. Consequently, the amount of gas produced did not reach the critical value required for bubble nucleation and growth, leaving the material relatively dense at this stage. A higher temperature is needed to accelerate the reaction of the foaming agent. Subsequently, at temperatures above 1050 °C, the process of pores expanding and growing was clearly observed. As the temperature rises, the number of pores decreases and the pore size process is illustrated in Figure 16.

### 3.7. EDS

**Figure 17:**
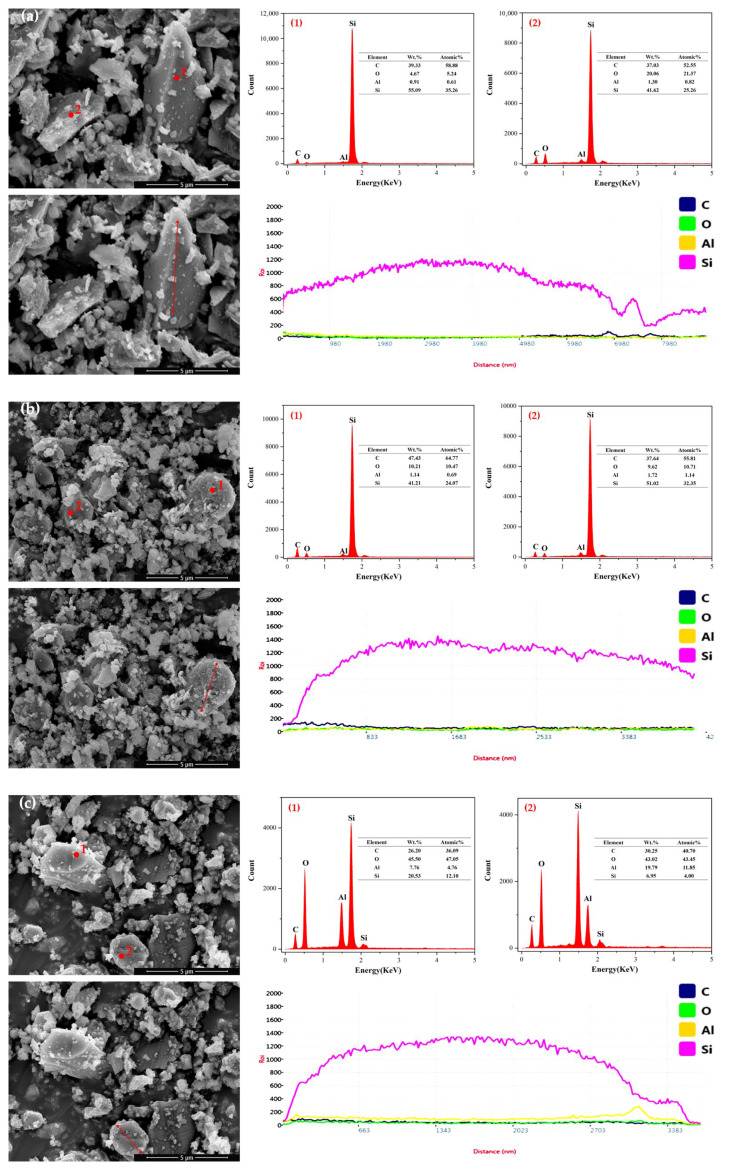
SEM and EDS of SiC with different ball milling methods ((**a**): untreated; (**b**): dry-grinding for 60 min; (**c**): wet-grinding for 60 min).

To further investigate the oxidation phenomenon of silicon carbide foaming agents during wet ball milling, In group A1, a certain amount of distilled water was added, the weight ratio of water to material was 1:2, polyvinyl alcohol with a concentration of 1% was added as the thickener, and the addition amount was 5% of the water content, which was placed in the ball mill tank for wet ball milling the surface elemental distribution of silicon carbide particles subjected to wet and dry ball milling for 1 h was analyzed, with the surface elements of untreated original SiC particles serving as the reference. Energy Dispersion Spectrum (EDS) was used to detect the types and contents of elements on the surface of the three silicon carbide samples. The EDS analysis results were shown in Figure 17.

EDS results indicated that the mass percentage of oxygen in the dry ball milled SiC was slightly higher than that of untreated SiC, while the mass percentage of silicon is slightly lower. This was attributed to the fact that the SiC was still oxidized in a certain degree during the dry ball milling process, which come from the heat generated by the milling and the minor oxygen content within the ball mill. After wet ball milling, the changes in elemental composition were more noticeable, with a remarkable decrease in silicon content and a significant increase in oxygen and aluminum content. This was due to the presence of water during the wet ball milling process, which might accelerate oxidation reactions. Additionally, the main material of the ball mill jar was Al_2_O_3_, which might be introduced as impurities during the milling process and lead to an increase in the mass percentages of oxygen and aluminum.

**Figure 18:**
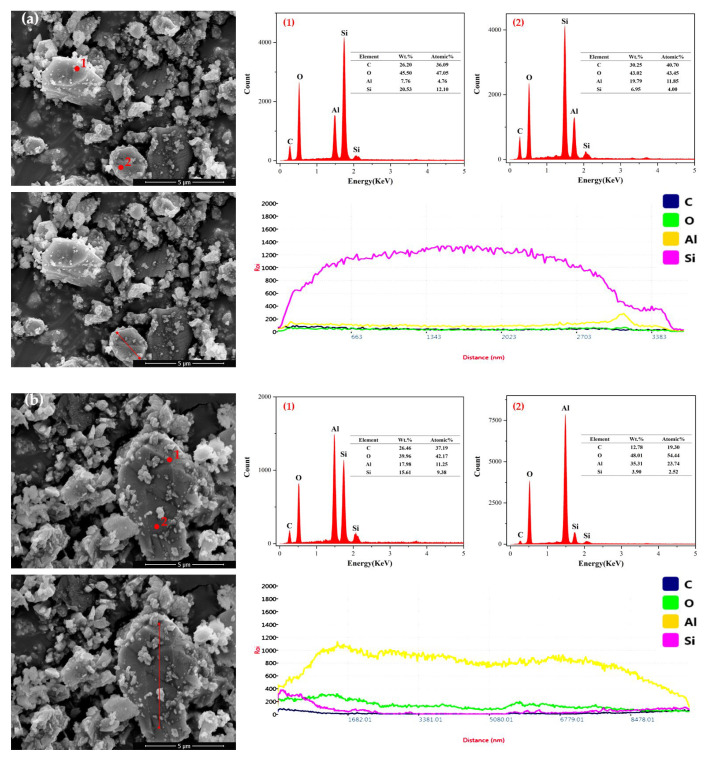
SEM and EDS of SiC at different ball milling times ((**a**): 60 min; (**b**): 120 min).

Considering the effect of ball milling time on the oxidation degree of SiC during wet ball milling, the samples after wet ball milling for 60 min and 120 min were dried, and surface elemental analysis was performed on the dried SiC particles. The results are shown in Figure 18. The EDS results indicated that the surfaces of both SiC particles contained four elements: C, O, Al, and Si. This was due to the fact that during the ball milling process, the finer SiC particles increased the surface energy and the reactivity, made it more susceptible to oxidation and led the increased oxygen content. Both. The aluminum content on the particle surfaces was from the ball milling container and grinding balls was made of Al_2_O_3_, the ball milling time has a significant effect on the oxidation of SiC particle surfaces, thereby influencing the reaction efficiency of the foaming agent in subsequent processes. According to the experimental results, a ball milling time of 60 min was suitable for the wet powder preparation process.

**Figure 19:**
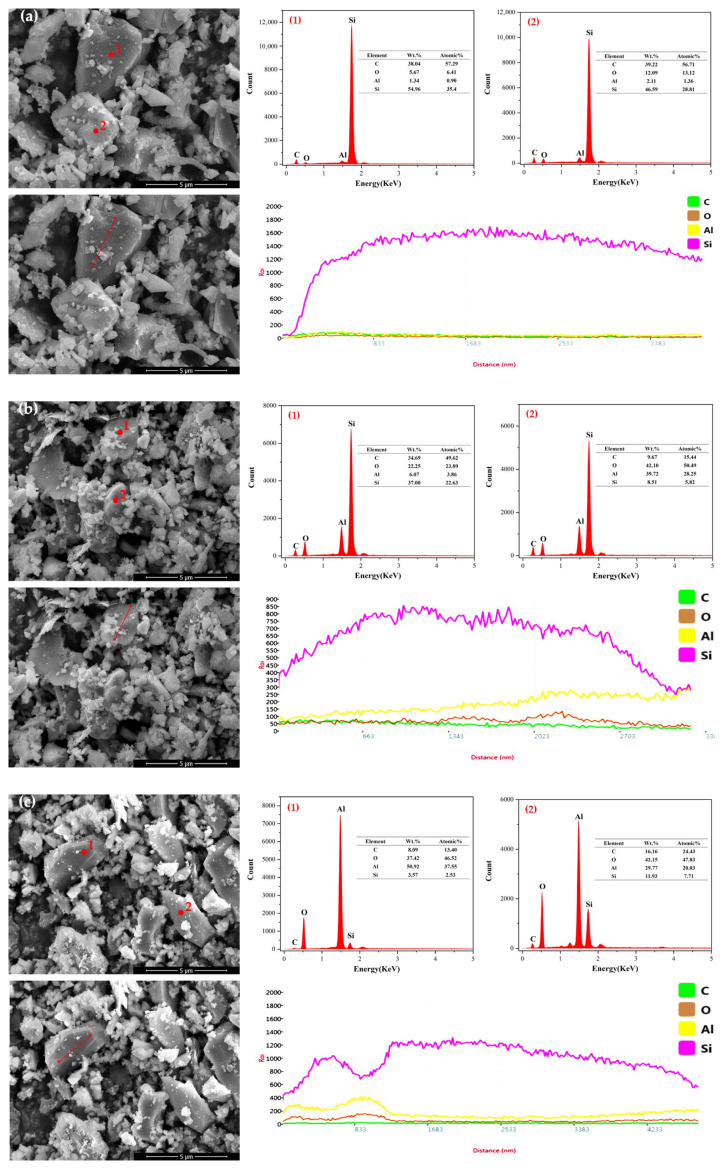
SEM and EDS of SiC at different drying times ((**a**): 30 min; (**b**): 60 min; (**c**): 120 min) (continued).

During wet ball milling, the drying time also affected the degree of oxidation of silicon carbide particles. Silicon carbide powders that underwent wet ball milling for 60 min were subsequently dried at 100 °C for 30, 60, and 120 min, respectively. The element types and contents on the three silicon carbide particles surfaces were analyzed using Energy Dispersive Spectroscopy (EDS) as shown in Figure 19 The EDS results indicated that at 30 min of drying, the oxygen (O) content on the particle surfaces was relatively low. At 60 min of drying, the O content significantly increased. And at 120 min of drying, the rate of increase in O content slowed down, with the content being slightly higher than at 60 min. This suggested that during the drying process of wet ball milling, the oxidation of silicon carbide particle surfaces became more noticeable around 60 min, followed by a slower rate of oxidation thereafter.

In summary, both ball milling methods caused varying degrees of oxidation on SiC, and wet milling resulted in a higher degree of oxidation. The impact of the wet grinding process on the oxidation of SiC primarily involved an increase in the foaming temperature of the material. At an appropriate foaming temperature, an ideal pore microstructure could still be obtained. In practical applications, it was advisable to select appropriate ball milling methods and process parameters based on specific requirements to minimize the oxidation of the foaming agent and enhance the performance of the porous ceramic materials.

## 4. Conclusions

This study utilized gold tailings as the primary raw material to prepare porous ceramic materials. It investigated the effects of factors such as gold tailings content, foaming temperature, raw material particle size, and foaming agent dosage on the microstructure, strength, thermal conductivity, and other properties of the porous materials. Additionally, it explored the impact of powder preparation methods on the oxidation degree of Sic foaming agents, and conducted an in-depth study on the intrinsic relationships between material composition, structure, and properties.

The increase in the usage of gold tailings could raise the temperature at which the system becomes liquid-phase and increasing the high-temperature viscosity of the melt. This made bubble formation and growth difficult at high temperatures, ultimately resulting in reduced pore size, lower porosity, higher bulk density, and increased thermal conductivity.

The sintering temperature had a significant impact on the reaction degree of the foaming agent and the liquid viscosity of porous ceramics made from gold tailings, thereby exerting a notable influence on the pore morphology. As the temperature increases from 1000 °C to 1090 °C, the microstructure of the samples underwent significant changes. At 1000 °C, there was a minor liquid phase, which made it difficult for the foaming agent to function effectively, resulting in low porosity within the materials. Only at 1050 °C did the materials begin to exhibit more noticeable pores internally. However, at excessively high temperatures of 1090 °C, the pores coalesced, leading to reduced uniformity.

The particle size of raw materials also affected the microstructure of porous ceramics made from gold tailings. The smaller the particle size of gold tailings, the higher the surface energy, which made it easier to melt and form the liquid phase. which facilitated bubble formation and increases porosity. Similarly, smaller SiC particles provided more nucleation sites for foaming, leading to the formation of more closed pores at high temperatures and further enhancing porosity.

In the preparation of porous ceramics from gold tailings, SiC foaming agent played a crucial role in pore formation. When the SiC content was low, the generated gas was insufficient to support the full growth of pores, resulting in smaller pore sizes and higher material bulk densities. As the SiC content increased, the amount of generated gas also increased. However, excessive usage of SiC led to an overproduction of gas, which promoted bubble growth and enhanced their mutual merging to form large pores. This caused changes in the pore structure and affected the material’s porosity and bulk density.

The production process of powder had a significant impact on the pore morphology of porous ceramics. Although wet production process of powder ensured uniform mixing, it could lead to oxidation of the silicon carbide surface during ball milling and drying, thereby increasing the foaming temperature. The porous ceramics prepared by the wet process exhibited more uniform pore sizes and superior microstructures, which were beneficial for reducing the thermal conductivity of the material and enhancing its strength.

Novelty: (a) This study utilized gold tailings as the primary raw material and silicon carbide micro powder as a high-temperature foaming agent to prepare porous ceramic materials. It investigated the effects of factors such as gold tailings content, foaming temperature, raw material particle size, and foaming agent dosage on the microstructure, strength, thermal conductivity, and other properties of the porous materials. Additionally, it explored the impact of powder preparation methods on the oxidation degree of Sic foaming agents, and conducted an in-depth study on the intrinsic relationships between material composition, structure, and properties.

(b) Further, this paper studied the chemical reaction of the silicon carbide foaming agent at high temperatures, observed the formation process of pores and their impact on the microstructure of the materials, and also explored the influence mechanism of the microstructure of materials on thermal conductivity.

Applications: While the title and keywords already indicate our focus on “Thermal Insulation,” we have reinforced this in the Conclusion by stating that the optimized materials are “highly competitive for insulation applications.”

Future Work: We have added a forward-looking perspective in the Author Contributions and Funding statements, indicating the ongoing nature of the research. A more specific recommendation for future work has been integrated into the final paragraph of the Conclusion, suggesting that “future work should focus on scale-up feasibility and long-term durability testing under simulated application conditions.”

## Figures and Tables

**Table 1:** Raw material formulation table of all samples.

Group Number	Gold Tailings (wt.%)	Perlite (wt.%)	Glass Powder (wt.%)	Boric Acid	SiC2#	Firing Temperature
A1	50	35	15	+2	+1	1050 °C
A2	60	25	15	+2	+1	1050 °C
A3	70	15	15	+2	+1	1050 °C
A4	80	5	15	+2	+1	1050 °C
A5	50	35	15	+2	+1	1060 °C
A6	50	35	15	+2	+1	1070 °C
A7	50	35	15	+2	+1	1080 °C
C1	50	35	15	+2	+0.0	1050 °C
C2	50	35	15	+2	+0.1	1050 °C
C3	50	35	15	+2	+0.2	1050 °C
C4	50	35	15	+2	+0.3	1050 °C

**Table 2:** Major oxide composition of raw materials (wt%).

	SiO_2_	Al_2_O_3_	K_2_O	Na_2_O	Fe_2_O_3_	MgO	CaO	SO_3_	Others
Gold tailings	61.60	13.50	6.60	1.95	5.06	2.52	6.01	1.67	1.09
Perlite	72.50	14.2	5.95	3.65	1.49	0.11	1.14	0.04	0.92
Glass powder	70.40	1.52	0.50	13.50	0.23	3.70	9.60	0.27	0.27

**Table 3:** Particle size of raw materials.

	B1	B2	B3	B4
Gold tailings (μm)	58	5.6	5.6	58
SiC (μm)	28.7	28.7	3.0	3.0

**Table 4:** Density and thermal conductivity of compact ceramics with different tailings amounts.

Content (%)	50	60	70	80
Bulk density (g/cm^3^)	2.06	2.21	2.22	2.24
Thermal conductivity (W/(m·k))	1.26	1.46	1.50	1.53

## Data Availability

The original contributions presented in this study are included in the article. Further inquiries can be directed to the corresponding author.
